# *Bombus (Pyrobombus) johanseni* Sladen, 1919, a valid North American bumble bee species, with a new synonymy and comparisons to other “red-banded” bumble bee species in North America (Hymenoptera, Apidae, Bombini)

**DOI:** 10.3897/zookeys.984.55816

**Published:** 2020-11-04

**Authors:** Cory S. Sheffield, Ryan Oram, Jennifer M. Heron

**Affiliations:** 1 Royal Saskatchewan Museum, 2340 Albert Street, Regina, Saskatchewan, Canada S4P 2V7 Royal Saskatchewan Museum Regina Canada; 2 British Columbia Ministry of Environment and Climate Change Strategy, Vancouver, British Columbia, Canada V6T 1Z1 British Columbia Ministry of Environment and Climate Change Strategy Vancouver Canada

**Keywords:** Arctic, bumble bee, DNA barcode, Holarctic species, melanism, morphology, synonymy

## Abstract

The bumble bee (Hymenoptera, Apidae, Bombini, *Bombus* Latreille) fauna of the Nearctic and Palearctic regions are considered well known, with a few species occurring in both regions (i.e., with a Holarctic distribution), but much of the Arctic, especially in North America, remains undersampled or unsurveyed. Several bumble bee taxa have been described from northern North America, these considered either valid species or placed into synonymy with other taxa. However, some of these synonymies were made under the assumption of variable hair colour only, without detailed examination of other morphological characters (e.g., male genitalia, hidden sterna), and without the aid of molecular data. Recently, *Bombus interacti* Martinet, Brasero & Rasmont, 2019 was described from Alaska where it is considered endemic; based on both morphological and molecular data, it was considered a taxon distinct from *B. lapponicus* (Fabricius, 1793). *Bombus interacti* was also considered distinct from *B. gelidus* Cresson, 1878, a taxon from Alaska surmised to be a melanistic form of *B. lapponicus sylvicola* Kirby, 1837, the North American subspecies (Martinet et al. 2019). Unfortunately, Martinet et al. (2019) did not have DNA barcode sequences (COI) for females of *B. interacti*, but molecular data for a melanistic female specimen matching the DNA barcode sequence of the holotype of *B. interacti* have been available in the Barcodes of Life Data System (BOLD) since 2011. Since then, additional specimens have been obtained from across northern North America. Also unfortunate was that B. sylvicola var. johanseni Sladen, 1919, another melanistic taxon described from far northern Canada, was not considered.

*Bombus johanseni* is here recognized as a distinct taxon from *B. lapponicus sylvicola* Kirby, 1837 (*sensu*Martinet et al. 2019) in the Nearctic region, showing the closest affinity to *B. glacialis* Friese, 1902 of the Old World. As the holotype male of *B. interacti* is genetically identical to material identified here as *B. johanseni*, it is placed into synonymy. Thus, we consider *B. johanseni* a widespread species occurring across arctic and subarctic North America in which most females are dark, with rarer pale forms (i.e., “*interacti*”) occurring in and seemingly restricted to Alaska. In addition to *B. johanseni* showing molecular affinities to *B. glacialis* of the Old World, both taxa also inhabit similar habitats in the arctic areas of both Nearctic and Palearctic, respectively. It is also likely that many of the specimens identified as *B. lapponicus sylvicola* from far northern Canada and Alaska might actually be *B. johanseni*, so that should be considered for future studies of taxonomy, distribution, and conservation assessment of North American bumble bees.

## Introduction

The bumble bees, *Bombus* Latreille, 1802 (Hymenoptera: Apoidea, Apidae) are one of the most thoroughly studied groups of bees, and extensive taxonomic coverage has existed for the North American fauna since Cresson (1863) first reviewed the species, with many subsequent taxonomic works (e.g., Franklin 1912; Frison 1923; Frison 1929; Lutz 1916; Lutz and Cockerell 1920; Stephen 1957; Thorp et al. 1983; Laverty and Harder 1988). In the most recent taxonomic treatment of bumble bees in North American north of Mexico, Williams et al. (2014) recognized 46 species, with two species of the subgenus Psithyrus Lepeletier, 1833 previously recognized as Nearctic, *B. fernaldae* (Franklin, 1911) and *B. ashtoni* (Cresson, 1864) considered synonyms of *B. flavidus* Eversmann, 1852 and *B. bohemicus* (Seidl, 1838), respectively; the latter two species thus treated as Holarctic. Since Williams et al. (2014), other taxonomic works have been published on the North American bumble bee fauna: in the subgenus Bombus, a subspecies of *B. occidentalis* Greene, 1858 was recognized, *B. occidentalis mckayi* Ashmead, 1902 (Williams et al. 2012; Sheffield et al. 2016); a new species of *Alpinobombus* Skorikov, 1914, *B. kluanensis* Williams & Cannings, 2016 was described from the Yukon and Alaska (Williams et al. 2016); and two additional species of *Alpinobombus*, *B. natvigi* Richards, 1931 and *B. kirbiellus* Curtis, 1835 were considered distinct from their Old World conspecifics, *B. hyperboreus* Schönherr, 1809 and *B. balteatus* Dahlbom, 1832, respectively (Williams et al. 2019). Within the subgenus Pyrobombus Dalla Torre, 1880, two taxa, *B. vancouverensis vancouverensis* Cresson, 1878 and *B. vancouverensis nearcticus* Handlirsch, 1888 were recognized as molecularly distinct from *B. bifarius* Cresson, 1878 (Ghisbain et al. 2020); *B. sylvicola* Kirby, 1837 was recognized as a subspecies of the Holarctic *B. lapponicus* (Fabricius, 1793); and a new species of *Pyrobombus* with close affinities to *B. lapponicus*, *B. interacti* Martinet, Brasero & Rasmont, 2019 was described from Alaska (Martinet et al. 2019). At this point, 48 species of bumble bee are now recognized in North America north of Mexico, though the taxonomic status of some species is still unresolved (e.g., Yanega 2013; Koch et al. 2018).

One common trend exists for most of these recently treated bumble bee species in North America – they are taxa with ranges that extend into, or are restricted to, northern regions of the globe. In North America and elsewhere, northern latitudes have been one of the most poorly studied and sampled regions for bumble bees (Potapov et al. 2019). Though the bee fauna of this region is typically considered much less speciose than others (for Canada, see Sheffield et al. 2014), it is of interest because of the obvious connection to the Old World via Beringia (Williams 1985; Hines et al. 2006; Williams et al. 2019).

Recent research contributing to the overall creation of a DNA barcode library for bees in Canada (Sheffield et al. 2017) was built on previous studies on the taxonomy and distribution of species in that country. For instance, Sheffield et al. (2011) recognized two *Megachile* species as Holarctic for the first time, and associated sexes for other species with the aid of DNA barcoding. In addition, DNA barcoding facilitated synonymies of taxa that were determined to be melanistic forms of other species (Sheffield et al. 2011), and the recognition of distinct taxa among cryptic species groups (e.g., Rehan and Sheffield 2011; Vickruck et al. 2012; Williams et al. 2012; Sheffield et al. 2016). Our main purpose here is to clarify the taxonomic status of a melanistic northern bumble bee taxon with close molecular and morphological affinities to an Old World taxon. A second objective is to provide diagnoses with accompanying photographs to facilitate identification of “red-banded” bumble bee species (i.e., Figure 5 “O” in Williams 2007; “Mimicry Pattern 5” of Williams et al. 2014) of North America in the field and from pinned specimens.

## Materials and methods

Bumble bee specimens from northern Canada contained the Royal Saskatchewan Museum (**RSKM**) were subject to DNA barcoding (Hebert et al. 2003) following procedures previously published for bees in Canada (Sheffield et al. 2009, 2017); all sequences used or created here and their associated specimen data are accessible through the Barcodes of Life Data System (BOLD) (Ratnasingham and Hebert 2007); Process IDs and Barcode Index Numbers (BINs; Ratnasingham and Hebert 2013) of specimens are provided in Table 1. Mitochondrial cytochrome oxidase subunit 1 (COI) gene sequences were obtained from samples of these and other pinned bumble bees of the subgenus Pyrobombus, and sequences from B. (Bombus) terricola Kirby, 1837 were selected as an outgroup; protocols for DNA extraction, polymerase chain reaction and sequencing follow those described elsewhere (Sheffield et al. 2009, 2017). Additional COI sequences were downloaded directly from GenBank (from Martinet et al. 2019) or from GenBank via BOLD (from Gjershaug et al. 2013, and Potapov et al. 2017) corresponding to *B. interacti* (male holotype only), *B. monticola* Smith, 1849, *B. lapponicus lapponicus*, and B. *glacialis* Friese, 1902, respectively (Table 2). These COI sequences were aligned with sequences in BOLD (MUSCLE) to provide confirmation of identification, and the reported genetic distances were analyzed using various sequence analysis tools on BOLD, including the Taxon ID Tree, Distance Summary, Barcode Gap Analysis, and Diagnostic Characters tools. Sequences were then downloaded as fasta (.fas) files and uploaded into MEGA X (Kumar et al. 2018) for phylogenetic analysis. All sequences were aligned using ClustalW, and the best DNA model using maximum likelihood was found based on the BIC value; the General Time Reversible with Gamma Variation (GTR+G) model was selected and a maximum likelihood tree was constructed with 500 bootstraps. Based on results of the tree, each taxon was collapsed and distance values within each (Tables 1, 2) were calculated to support species group delineation. Distances among taxa were also calculated, and diagnostic nucleotides for species of interest were determined (Table 3).

**Table 1.** Species and specimens of bumble bees with COI sequences in BOLD used for genetic analyses in this study, including BOLD Process IDs (when available) for each specimen, the Barcode Index Number (BIN) to which the specimens have been assigned, and the genetic distance observed within each species.

Species	BOLD Process ID	BIN	Genetic distance (%)
*Bombus johanseni*	ACHAR117-18, CCHAR061-19, CCHAR062-19, ACHAR3482-19 , DCHAR2640-19, MOBIL1097-15, BEECF718-11, WASPS1609-20, WASPS1607-20, WASPS1603-20, WASPS1604-20, WASPS1605-20, FCHAR3225-19, FCHAR4872-19, FCHAR4878-19	BOLD:ABA8452	0.0009
*B. bimaculatus*	UPOLB204-09, UPOLB218-09, BOWGF1488-10, BEECD873-10, BOWGF1653-10	BOLD:AAB4829	0
*B. sylvicola* s. str.	BEECA042-06, BEECA296-06, BEECA297-06, JSYKA168-10, JSYKA176-10, BWTWO1164-10	BOLD:AAA8078	0
*B. melanopygus*	TTHYW305-08, TTHYW340-08, TTHYW341-08, TTHYW479-08, BCLRB862-10, BEECD829-10, BCII522-10, BCII742-10, BCII743-10	BOLD:AAB5223	0.0004
*B. ternarius*	BEECD864-10, BEECD865-10, BEECD867-10, BBHYL228-10, OPPFC190-17, OPPFE004-17	BOLD:AAB5221	0.0022
*B. perplexus*	TTHYW593-08, TTHYW595-08, TTHYW616-08, BBHEC177-09, BEECD417-09, BEECD419-09, BEECD422-09, BEECD423-09, BEECD424-09, BEECD436-09, BEECD878-10, BEECD879-10	BOLD:AAB2150	0.0012
*B. sitkensis*	TTHYW283-08, BCII485-10, BCII486-10, BBHYL258-10, BCIII001-11	BOLD:AAI4757	0.003
*B. mixtus*	BCLRB866-10, BCLRB920-10	BOLD:AAB1091	0
*B. jonellus*	BEECF862-12, BEECF863-12, BEECF873-12, BEECF887-12, BOWGF2140-12, UAMIC749-13	BOLD:AAD4941	0.0135
*B. frigidus*	MHBEE033-07, MHBEE034-07, TTHYW237-08, BWTWO1201-10, BEECE682-10, BEECE715-10, BBHYL221-10	BOLD:AAB1090	0.001
*B. flavifrons*	BEECA039-06, BEECA040-06, HMBCH001-07, TTHYW207-08, TTHYW234-08, TTHYW313-08, TTHYW488-08, BOWGF787-09, JSYKA173-10, JSYKA174-10, BBHYL273-10	BOLD:ACE3465	0.0011
*B. terricola*	TTHYW654-08, TTHYW807-08, BEECD330-09, BEECD331-09, BBHEC139-09, BBHEC143-09, BBHEC144-09, BWTWO706-09, BWTWO707-09, BEECD383-09, BEECD410-09, BEECD735-09, BCLRB868-10	BOLD:AAA8658	0.0001

To document the geographic range of the taxon of interest, localities from literature (i.e., Cresson 1878; Ashmead 1902; Franklin 1912; Sladen 1919; Bequaert 1920; Lutz and Cockerell 1920; Frison 1927a; Martinet et al. 2019) and from specimens at the RSKM were mapped using SimpleMappr (Shorthouse 2010) based on original taxon name and colour pattern. In addition, records from iNaturalist (www.inaturalist.org) were first verified, with data subsequently mined. The full dataset for the specimens used in this study is archived with Canadensys (http://community.canadensys.net/) under resource title “*Bombus johanseni*, a valid North American bumble bee species” and can be accessed using the following: https://doi.org/10.5886/3ex36t.

**Table 2.** List of *Bombus* species for which sequences were obtained from GenBank, with GenBank accession numbers, Barcode Index Number (BIN) and the genetic distance observed within each species. Published source of the data are Gjershaug et al. 2013 (*Bombus monticola, B. lapponicus*); Potapov et al. 2017 (*B. glacialis*); Martinet et al. 2019 (*B. interacti*). *see *B. johanseni* in Table 1.

Species	GenBank Acc. No.	BIN	Genetic distance (%)
*B. glacialis*	KY202838, KY202839, KY202840, KY202841, KY202842, KY202843	BOLD:ADU5113	0
*B. interacti*	MG280603	BOLD:ABA8452	*
*B. monticola*	GU705913, KJ838349, KJ838456, KJ837131, KF434337, KF434338, KF434339	BOLD:AAD8242	0.0011
*B. lapponicus*	KF434329, KF434330, KF434331, KF434332	BOLD:AAA8078	0.0011

Photomicrography was undertaken with a Canon EOS 5D Mark II digital camera with an MP-E 65 mm 1:2.8 1–5× macro lens. Measurements were made with an ocular micrometer on a Nikon SMZ1000 stereomicroscope.

**Table 3.** Diagnostic nucleotides and their position within the COI mitochondrial gene (i.e., DNA barcode) for *Bombus glacialis* (ADU5113), *B. johanseni* (ABA8452, includes *B. interacti*), *B. lapponicus* (ssp. lapponicus – AAA8078), *B. monticola* (AAD8242) and *B. lapponicus* (ssp. sylvicola – AAA8078).

Species	Nucleotide position																						
	48	105	195	207	241	259	270	318	333	334	349	387	402	411	433	447	504	537	540	555	603	607	648
*B. glacialis*		C	C				A										C					C	
*B. johanseni*				A	C			C						C				C	C		G		
*B. lapponicus*	C					C																	
*B. monticola*								A		C	A	G	C			C							C
*B. sylvicola*									G						G					C			

## Results

**Figure 1.**
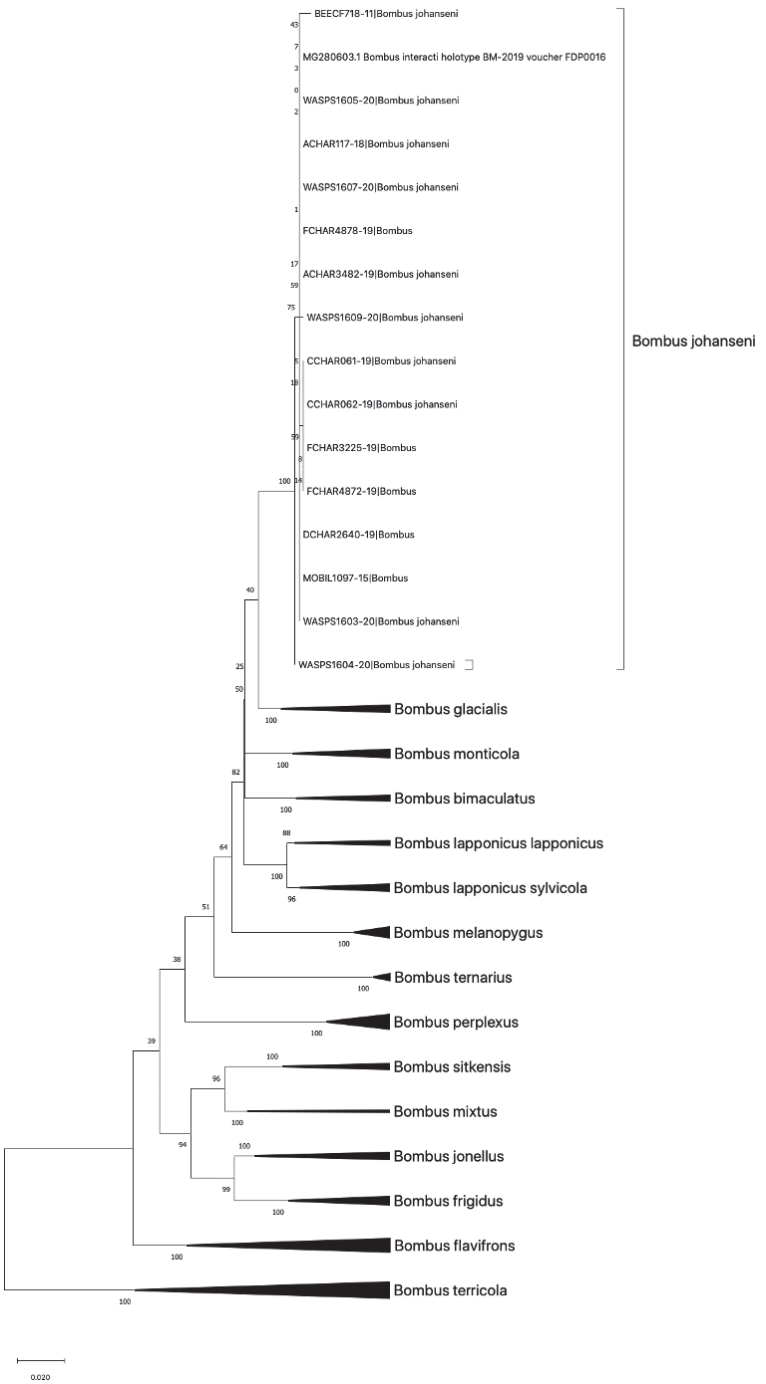
Maximum likelihood tree constructed with 500 bootstraps for selected taxa of *Pyrobombus*, with B. (Bombus) terricola as an outgroup. The taxon *Bombus johanseni* consists of specimens identified in BOLD as *Bombus* sp. and *B. johanseni*, in addition to the holotype male of *B. interacti*.

Phylogenetic analysis and genetic distance of COI sequences support the close affinity of *B. johanseni* to *B. glacialis* (1.98% genetic distance) and *B. monticola* (2.74% genetic distance), both Old World taxa (Fig. 1), and support that *B. johanseni* should not be considered conspecific with *B. lapponicus sylvicola* (3.49% distance). In addition, the COI sequence from the holotype male of *B. interacti* shows no differences from specimens matching the type material and descriptions *B. johanseni* (Fig. 1), all belonging to BIN ABA8452, with <0.001% genetic distance among specimens (Table 1), supporting the synonymy below.

### Taxonomic accounts

#### Bombus (Pyrobombus) johanseni

Taxon classificationAnimaliaHymenopteraApidae

Sladen, 1919 comb. nov.

C76E0117-BA5F-55B7-95EE-C34AD0A3A5DE

**Figure 2.**
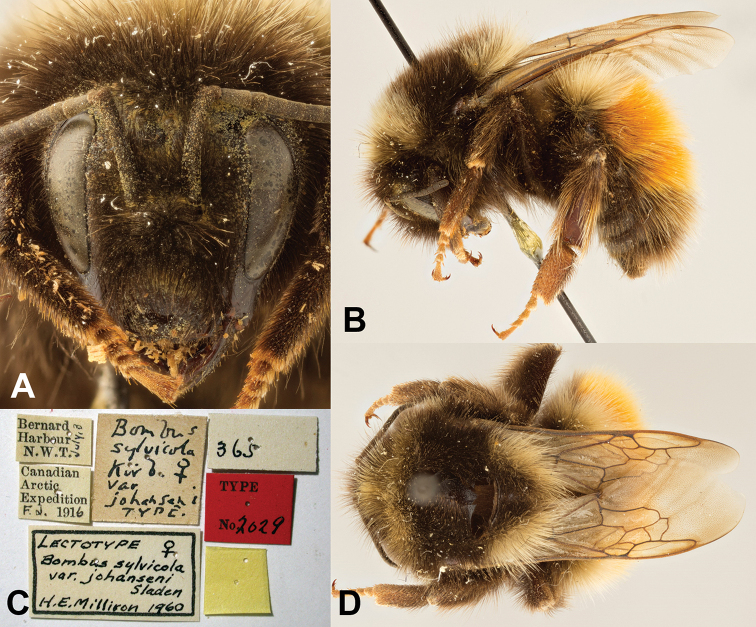
Holotype female of Bombus sylvicola var. johanseni**A** face **B** lateral view **C** associated specimen labels **D** dorsal view. Photographs by Joel Kits, Ottawa Research and Development Centre.

Bombus sylvicola var. johanseni Sladen, 1919: 30g [♀]. **Holoytpe** ♀. CANADA, Northwest Territories^*^, Bernard Harbour, 3 July 1916 [3 July 1915], Canadian Arctic Expedition, by F. Johansen [Canadian National Collection of Insects, Arachnids, and Nematodes, CNC no. 2029]. [photographs of holotype examined, see Fig. 2].
Bombus (Pyrobombus) interacti Martinet, Brasero, & Rasmont, 2019: 611 [♀, ♂]. syn. nov. **Holotype** ♂. USA, Alaska, Toolik field station, 68°37'32.9"N 149°35'48.8"W, 725m, 28 July 2015, by Martinet and Rasmont, on Epilobium angustifolium [Royal Belgian Institute of Natural Sciences]. Photographs of holotype (as per Martinet et al. 2019) examined.

##### Diagnosis.

**Figure 3.**
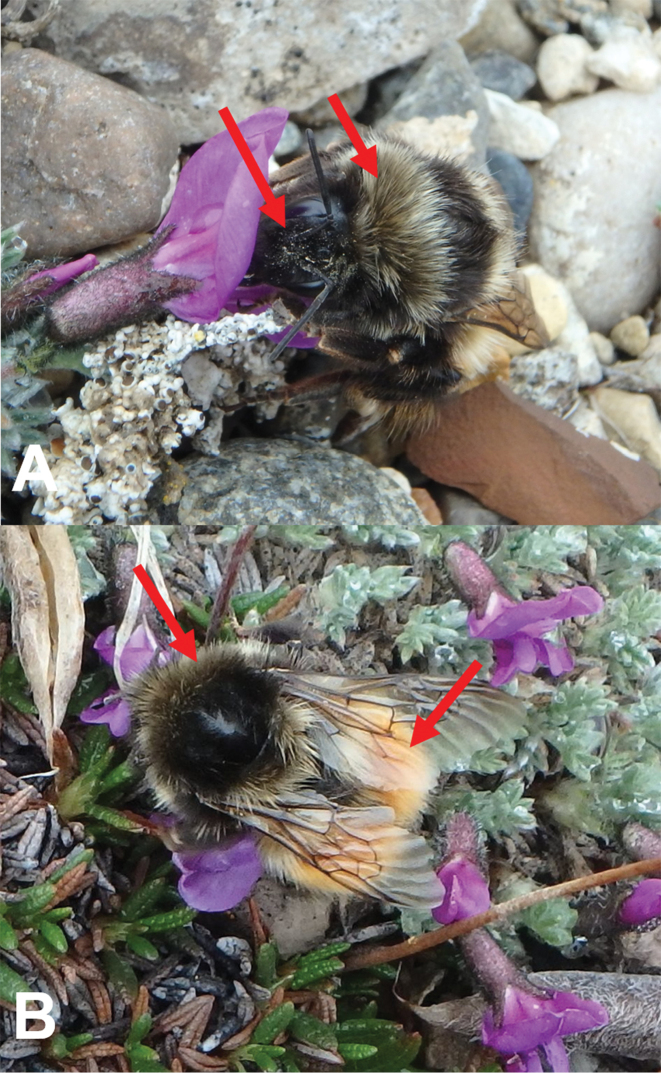
Bombus johanseni female from Sachs Harbour, Banks Island, Northwest Territories, Canada. **A** Left arrow shows the characteristic black pubescence of the face, right arrow shows the solid area of darker pubescence on the anterior part of thorax **B** left arrow shows the solid area of darker pubescence on the anterior part of thorax, right arrow shows the typical “red-banding” of the abdomen. Photographs by JMH.

**Figure 4.**
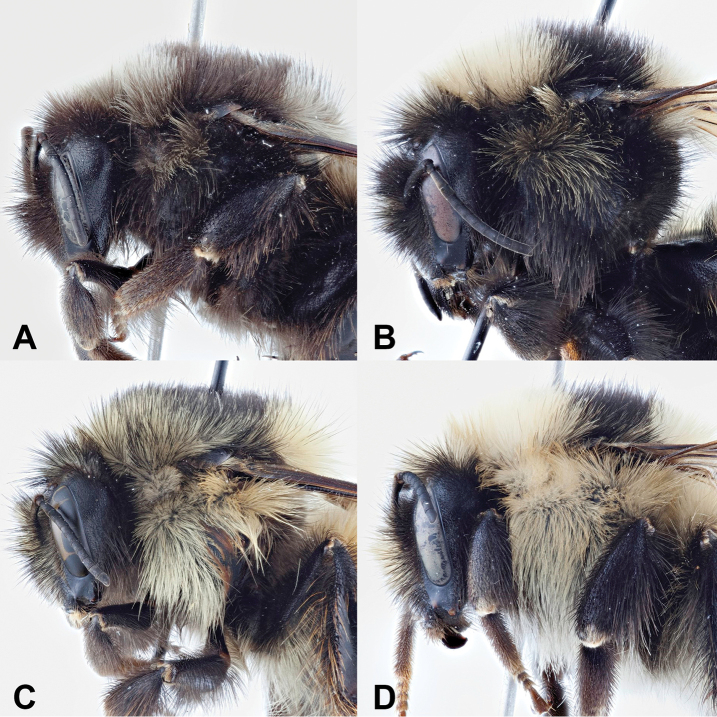
Lateral view of the thorax of female Bombus species. **A, B**Bombus johanseni**C**B. melanopygus**D**B. lapponicus sylvicola.

**Figure 5.**
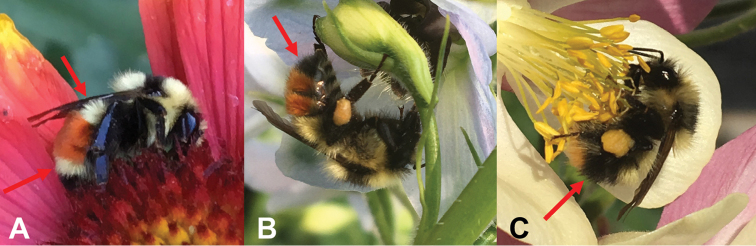
**A** A typical “red-banded” bumble bee, Bombus huntii, showing the two red bands (terga 2 and 3) with yellow bands on either side (arrows, terga 1 and 4), and “red-tailed” bumble bees **B**B. centralis, with red band (terga 3 and 4) preceded by a yellow band (terga 1 and 2) with black apically (arrow) **C**B. mixtus, with a black band (arrow) separating the basal yellow and apical red bands. Photographs by CSS.

**Figure 6.**
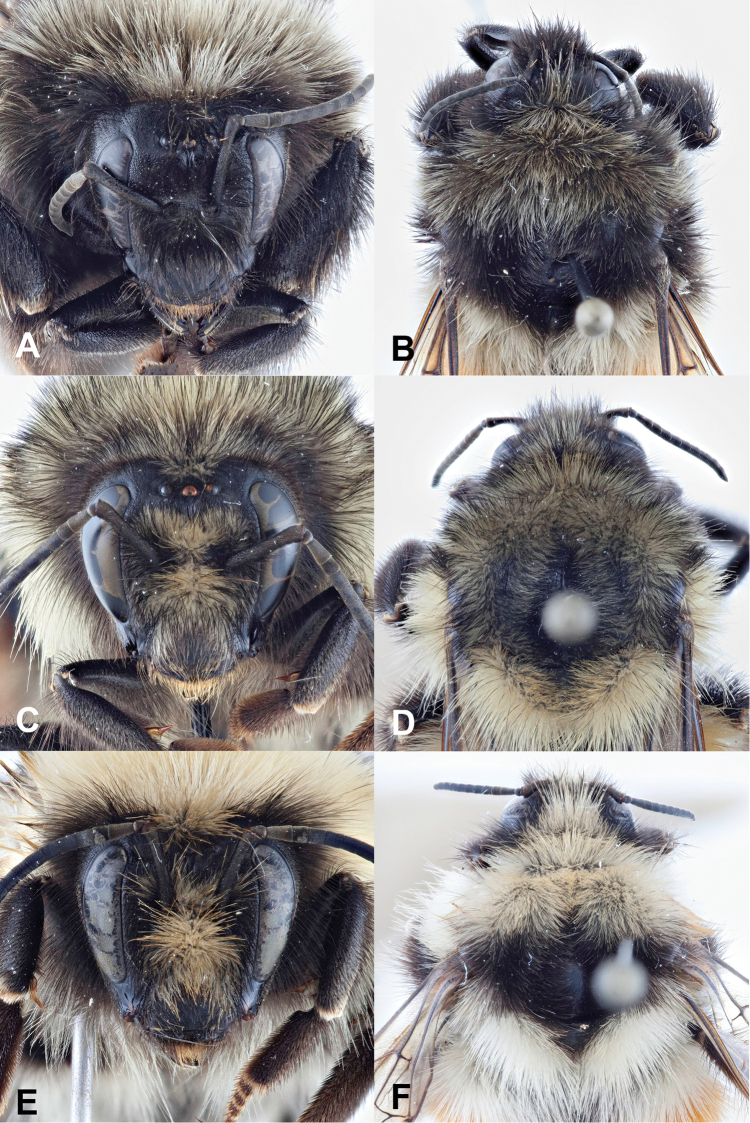
Faces (**A, C, E**) and thorax in dorsal view (**B, D, F**) in female bumble bees **A, B**Bombus johanseni**C, D**B. melanopygus**E, F**B. lapponicus sylvicola.

**Figure 7.**
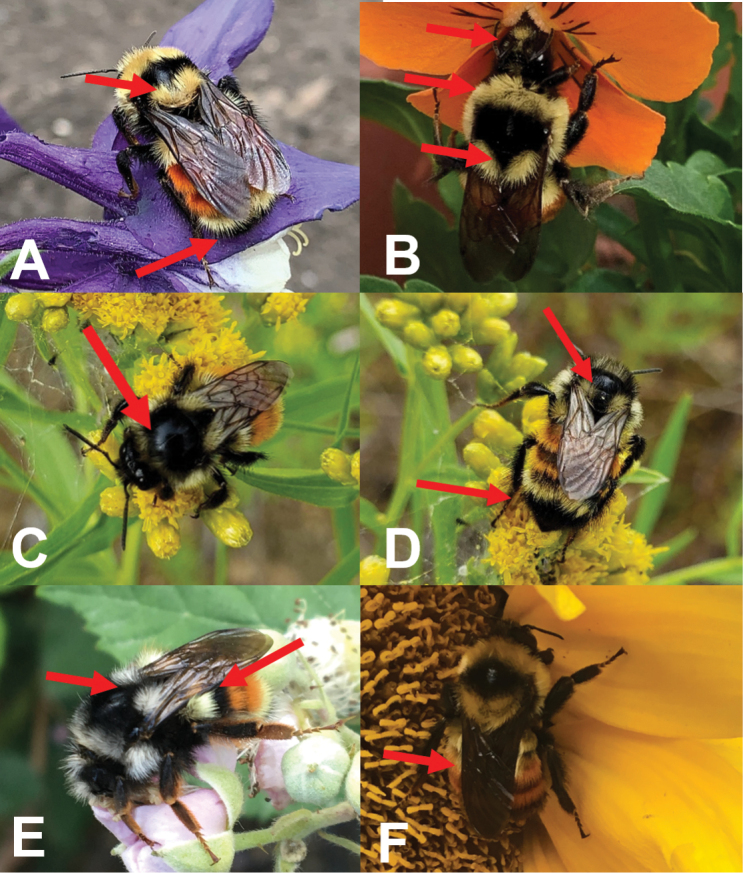
Examples of “red-banded” bumble bees. **A**Bombus huntii female. Top arrow shows the complete yellow hair patch on the rear of the thorax (scutellum), bottom arrow shows the completely black tegum 5 **B**B. ternarius female. Top arrow shows the face with both yellow and black hairs, middle arrow shows the entirely yellow anterior area of the thorax, bottom arrow shows the yellow hair patch on the rear of the thorax (scutellum) divided in two by a wedge of black hairs. Photographs by CSS **C, D**B. ternarius female, from Newfoundland. Arrow in **C** shows the atypical intermixed black hair on the anterior area of the thorax. Top arrow in **D** shows the yellow hair patch on the rear of the thorax (scutellum) divided in two by a wedge of black hairs, bottom arrow shows all black tergum 5. Photographs by Carolyn Parsons **E**B. vancouverensis female (red form). Left arrow shows the pale hair patch on the rear of the thorax (scutellum) divided in two by a wedge of black hairs, right arrow shows the incomplete red band of tergum 2, with black hairs in basal half **F**B. rufocinctus female (red form). Arrow shows the incomplete red band of tergum 2, being yellow medially in the basal half. Photographs by CSS.

Among the members of the Bombus lapponicus – complex, and other Pyrobombus considered here, B. johanseni is genetically most similar to the northern Palearctic B. glacialis (and see Martinet et al. 2019). In northern North America, the melanistic females of B. johanseni (Figs 2, 3, 4a, b) are most similar to darker forms of B. melanopygus (Fig. 4c) and atypical dark forms of B. ternarius Say, 1837 from Newfoundland and Labrador (Fig. 7c, d), while paler forms (i.e., “interacti”) are most similar to B. lapponicus sylvicola (Fig. 4d); all these taxa have the characteristic “red-banded” metasomal colour pattern of tergum 1 primarily yellow, terga 2 and 3 primarily red or orange, and tergum 4 primarily yellow at least laterally (Fig. 5a); with T5 yellow, at least laterally. Bombus johanseni females differ from these other taxa by the colour of the pubescence on the face, being entirely dark in B. johanseni (Figs 2a, 3a, 4a, b, 6a, b; but see Martinet et al. 2019), but primarily yellow in B. lapponicus sylvicola (Figs 4d, 6e) or strongly intermixed in B. melanopygus (Figs 4c, 6c). The dark forms of B. johanseni also have extensive dark pubescence on much of the mesosoma, including the pleura (Figs 2, 3, 4a, b), with the dark pubescence extending laterally on the dorsal anterior surface (Figs 2, 3a, 4a, 6a), the latter characteristic shared only with dark specimens of B. melanopygus, though in the latter species the hair is usually intermixed (Figs 4c, 6c, d), not a solid colour (Figs 2, 3, 4a, 6a, b). Other specimens of B. johanseni have dark hairs on the pleura, with hairs becoming paler on the dorsal surface (Fig. 4b), while others (i.e., interacti) are almost entirely pale haired on the pleura (but becoming somewhat darker below) and dorsal surface (see Martinet et al. 2019) and more closely resemble B. lapponicus sylvicola (Figs 4d, 6e, f). Morphologically, the females of B. johanseni and B. lapponicus sylvicola are very similar (Martinet et al. 2019), as are the Old World taxa B. glacialis and B. lapponicus lapponicus (Potapov et al. 2017).

The “red-banded” pattern (Fig. 5a) of these northern taxa is also shared with other, typically more southern species, including B. ternarius, B. huntii Greene, 1860, some B. vancouverensis, and some B. rufocinctus Cresson, 1863 (Fig. 7, and see Mimicry Pattern 5 in Williams et al. 2014), though these latter species generally tend to have females with either T5 entirely black (B. huntii, B. ternarius, Fig. 7a, d), or with tergum 2 black (B. vancouverensis, Fig. 7e) or yellow (B. rufocinctus, Fig. 7f) basiomedially.

**Figure 8.**
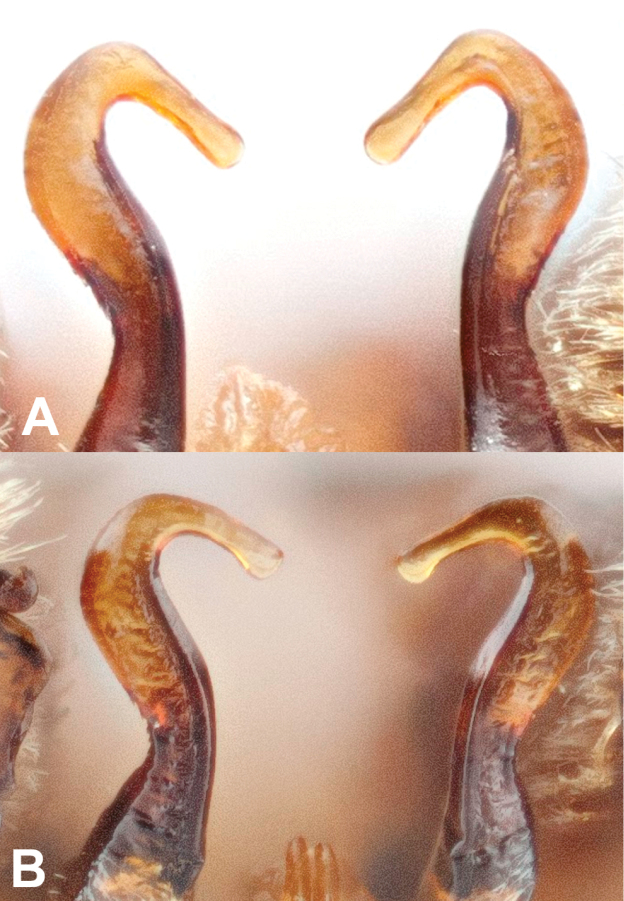
Penis-valve head of male **A**Bombus johanseni, and **B**B. lapponicus sylvicola.

**Figure 9.**
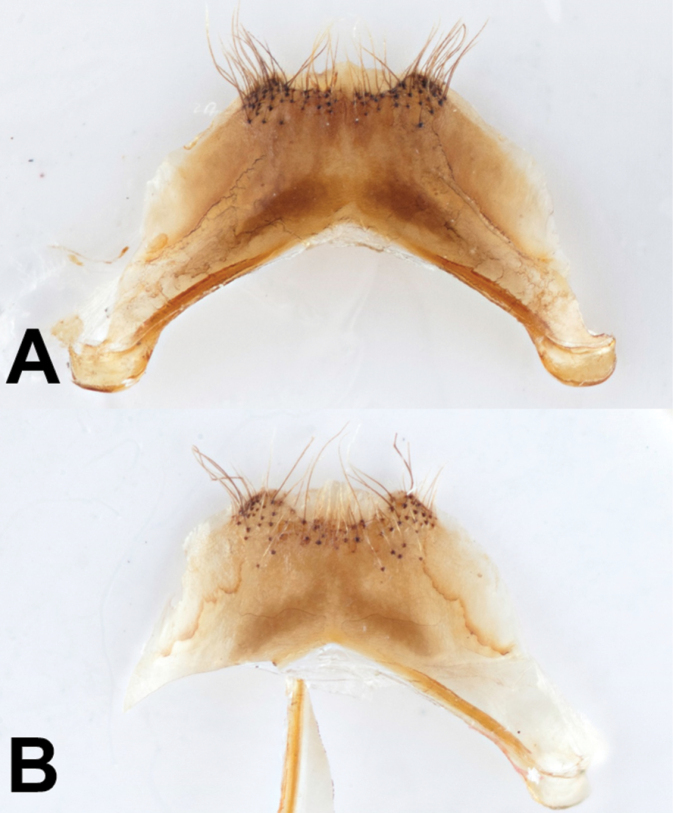
Sternum 7 of male **A**Bombus johanseni, and **B**B. lapponicus sylvicola.

In North America, the males of B. johanseni resemble B. lapponicus sylvicola, B. ternarius, B. huntii, some B. vancouverensis, and pale individuals of B. melanopygus. The males of B. johanseni and B. lapponicus sylvicola can be distinguished from all other Pyrobombus in North America by the bulbous tip of the penis valve (Stephen 1957; Thorp et al. 1983; Williams et al. 2014) (Fig. 8), though in the former, the tip of the penis valve (Fig. 8a) is not quite as bulbous as in B. lapponicus sylvicola (Fig. 8b). In B. johanseni, sternum 7 has more elongate hairs on the apicolateral edges, with a shallower apicomedial depression somewhat rectangular, approximately 1/4 as deep as wide (Fig. 9a), but broadly U-shaped in B. lapponicus sylvicola, and 1/3 as deep as wide (Fig. 9b).

## Discussion

The *Bombus lapponicus* – complex (Williams et al. 2014) has been of interest to many researchers in the Old World for some time, with many taxa recognized at subspecific rank (e.g., Pittioni 1942, 1943), but also as distinct species in the past (Svensson 1979; Pekkarinen 1982; Martinet et al. 2018) and more recently (Gjershaug et al. 2013; Potapov et al. 2017, 2019). In a recent treatment of the North American members, Martinet et al. (2019) recognized *B. lapponicus sylvicola* as a Nearctic subspecies, with the typical taxon occurring in the Palearctic, supporting previous speculation on the status of this species (e.g., Pittioni 1943, Thorp 1962; Thorp et al. 1983; Williams et al. 2014). Hines et al. (2006) indicated that this species group originated in the New World, with dispersal to the Old World occurring within the last 4 million years. Though colour variation in *B. lapponicus* in the Old World is substantial (see Gjershaug et al. 2013), resulting in the past recognition of many subspecific taxa (i.e., Pittioni 1943), it mostly retains the colour pattern typical of the Western Nearctic taxon in parts of its Old World range (Hines et al. 2006; Williams 2007; Martinet et al. 2019). Exceptions include the specimens originally described as *B. gelidus* Cresson, 1878 described from Alaska (Cresson 1878) and Bremus sylvicola var. lutzi Frison, 1923 described from Arizona (Frison 1923), both of which have the pleura with dark hairs in the lower half, and the face with hairs mostly black, with slight intermixtures of pale hair (Cresson 1878; Franklin 1912; Frison 1923, 1927a). In North America, the typical form of *B. lapponicus sylvicola* is widespread in boreal-alpine areas, including throughout most of the north and in western mountain regions, though another dark form with the red hairs of terga 2 and 3 of the typical form replaced by black hairs is found in the Sierra Mountains of California (Williams et al. 2014). The nature and ecological significance of melanism in bumble bees has been the subject of several studies (e.g., Williams 2007; Rapti et al. 2014; Polidori et al. 2017), and significant variation is common within and among species (Stephen 1957; Williams et al. 2014; Huang et al. 2015).

**Figure 10.**
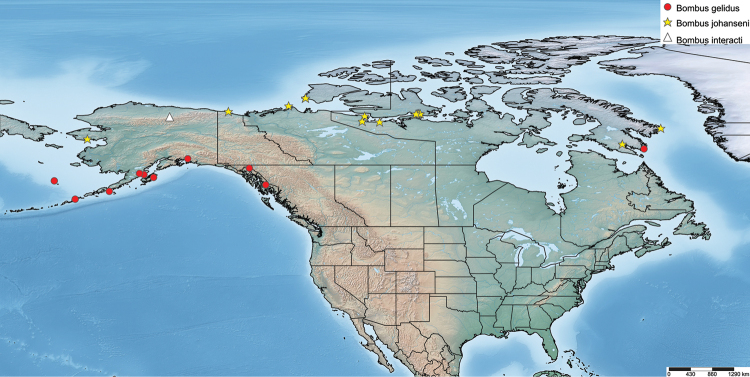
Distribution map of known specimens of *B. johanseni*, including *B. interacti*. Specimens identified in the literature as *B. gelidus* are also included as it is likely that some of these, especially the eastern record, are actually *B. johanseni*, and not dark specimens of *B. sylvicola*.

Martinet et al. (2019) also described another member of this species complex sharing this colour pattern, *B. interacti*, a species almost identical to *B. lapponicus sylvicola*, with mostly pale pleura in both sexes, and presumably with identical male genitalia; only slight morphological differences in pubescence colour and density were noted, in addition to the molecular and semio-chemical differences (Martinet et al. 2019). The differences in males of these two taxa, as diagnosed by Martinet et al. (2019), were based on the pubescence of the [hind] tibia, being “very hairy” in *B. lapponicus sylvicola* but presumably not so in the holotype male of *B. interacti*, the latter thus matching Franklin’s description of the male of *B. gelidus* (Franklin 1912); we now assume that what Franklin (1912) was describing was likely the male of *B. johanseni*/*B. interacti*. As reported by Sikes and Rykken (2020), *B. interacti* is considered very rare, representing one of almost 34,000 bumble bee records in that study; the type series contained ten males and four queens from Alaska (Martinet et al. 2019). Also, in that work, Martinet et al. (2019) supported the opinion of some earlier works (e.g., Franklin 1912; Frison 1927a, 1927b; Stephen 1957; Hurd 1979; Williams et al. 2014), but not all (Ashmead 1902; Lutz 1916; Lutz and Cockerell 1920) on the affinity of *B. gelidus* to *B. lapponicus sylvicola*, treating it as a synonym (i.e., as forma gelidus) and indicating that it was just a melanistic form. Williams et al. (2014) also mentioned *B. gelidus* as a dark form of *B. lapponicus sylvicola* (with dark hairs on the face and sides of the thorax) found most frequently in Alaska, but did not include a representative colour pattern to account for the variation in this species. *Bombus gelidus* was described from the Aleutian Islands of Alaska (Cresson 1878), albeit only from the single female (queen) type specimen that was examined by Martinet et al. (2019). Other materials identified as *B. gelidus* by T.D.A. Cockerell and incorrectly labelled as co-types by Franklin (1912) were typical *B. lapponicus sylvicola* according to Martinet et al. (2019); among this material was one additional queen, 14 workers, and a male. Like the holotype, these specimens were also from Alaska, including the Shumagin Islands group (Popoff Island), the Aleutian Isands (Nualaska, presumably Unalaska), and from the southern mainland (Koyukuk River, Kukak Bay) and collected by Trevor Kincaid during the Harriman Alaska Expedition in 1899. Ashmead (1902) originally published on the Hymenoptera collected during the Harriman Alaska Expedition, and in addition to identifying *B. gelidus* (after examining the holotype) from the Pribilof Islands, an island group much more isolated than the Aleutian Islands, he also (and incorrectly) synonymized *B. kincaidii* Cockerell, 1898 (=Bombus (Alpinobombus) polaris Curtis, 1835) under that species. Bequaert (1920) also identified *B. gelidus* from Alaska, from Kodiak, Katmai, and Valdez. An additional, albeit aberrant worker of *B. gelidus* was mentioned but not described by Franklin (1912) from Signuia, Baffin Island (Nunavut) (Fig. 10), which if correct would suggest that the melanistic form was more widespread than just Alaska (Williams et al. 2014; Martinet et al. 2019); an alternative and more likely explanation is that this aberrant specimen was what Sladen (1919) later named B. sylvicola var. johanseni. Similarly, Lutz (1916) recorded five additional specimens of *B. gelidus* from Battle Harbor, Labrador collected by C.W. Leng, but Lutz and Cockerell (1920) later determined these to be *B. ternarius*, typically a southern species in the east (Laverty and Harder 1988) though more recently found in Nunavut (Gibson et al. 2018). However, Packard (1891) recorded *Bombus lacustris* Cresson, 1863 (= *B. melanopygus*) as common on the northern coast of Labrador, though Frison (1926) suggested that these were likely *B. lapponicus sylvicola*, a species much more common in northern Labrador than *B. melanopygus* (Williams et al. 2014). As Cresson (1863) described *B. lacustris* as a taxon with much black hair intermixed with the yellow on the head and thorax, it is possible Packard (1891) observed *B. johanseni*, not *B. lapponicus sylvicola*, thus supporting its presence in Labrador. Another possibility is that these were atypical *B. ternarius*, as specimens from Labrador and insular Newfoundland typically have large intermixtures of black and yellow hair on the anterior thorax (Fig. 7c, d), thus resembling *B. melanopygus*.

Stephen (1957) also examined material identified as *B. gelidus* from Alaska, but indicated that the male genitalia and sterna 7 and 8 were similar to that of *B. melanopygus*, though he also felt that *B. lapponicus sylvicola* and *B. melanopygus* were virtually indistinguishable in parts of their ranges, so these specimens may have been misidentified. The male of *B. gelidus* was apparently known by Franklin (1912) and Bequaert (1920), the former offering a brief description, but was described in more detail by Martinet et al. (2019). The morphological characteristics used to distinguish *B. gelidus*/*B. lapponicus sylvicola* from *B. interacti* were subtle (Martinet et al. 2019).

Unfortunately, Sladen’s taxon B. sylvicola var. johanseni has received little attention, and until this time it was still considered conspecific with *B. lapponicus sylvicola* (Lutz and Cockerell 1920; Pittioni 1943; Hurd 1979), nor was it included in the treatments of Stephen (1957) and Thorp et al. (1983) for western North America, nor Laverty and Harder (1988) in the east. Though clearly described as a melanistic variety of *B. lapponicus sylvicola* (Sladen 1919), seemingly even much more so than *B. gelidus* (Figs 2, 4a, b; Martinet et al. 2019: fig. 7), with a distribution now known to range far into Canada’s eastern arctic region westward to Alaska (Fig. 10), Sladen’s taxon has largely been ignored. Genetic (Fig. 1) and morphological evidence provided here allies *B. johanseni* most closely with *B. glacialis*, a species with a similar northern distribution in the Old World (Potapov et al. 2017, 2019) and considered a valid species in the phylogenetic analysis of Martinet et al. (2019); both *B. johanseni* and *B. glacialis* are clearly genetically distinct (3.5% and 2.8%, respectively) from *B. lapponicuss. l.* (Fig. 1, and see Potapov et al. 2017 and Martinet et al. 2019). Molecular data (Fig. 1), the distinct colour patterning (Figs 2, 4, 6), male genitalia (Fig. 8) and sternum 7 (Fig. 9) provide evidence that *B. johanseni* is not a melanistic form of *B. lapponicus sylvicola*, but rather a distinct taxon with a northern Nearctic distribution. Additionally, *B. johanseni* males are seemingly morphologically similar to those of *B. interacti*, and also do not differ genetically (Fig. 1), supporting the synonymy above. Females of *B. johanseni* are also morphologically similar to dark females of *B. lapponicus sylvicola* (i.e., *B. gelidus*), though the latter taxon almost always has some pale hairs on the face and seems more common in southern Alaska (Fig. 10), though more sampling is required to determine the extent of this form. As *B. johanseni* is the oldest name available for this taxon, we here consider it a valid species, and as COI sequences from these darker taxa match the male holotype of *B. interacti*, we synonymize that species under *B. johanseni*, considering it a rarer (Fig. 10; and see Sikes and Rykken 2020) paler form. As shown by Huang et al. (2015) and Williams et al. (2019), species with wide variance in colour pattern may show little covariation in COI. At present, the darker forms of *B. johanseni* seem widespread across northern North America (Fig. 10), while the females of the pale form are seeming only observed, albeit rarely, in Alaska (Martinet et al. 2019; Sikes and Rykken 2020).

Future phylogenetic analysis that includes all New and Old World *Pyrobombus* may clarify the relationships between *B. johanseni* and *B. glacialis*, though it would be very useful to obtain additional material, including males, from the Aleutian Islands for additional molecular and morphological analyses. This island chain has proven an interesting link to the Old World bumble bee fauna (Williams and Thomas 2005, Sheffield and Williams 2011). Until a globally comprehensive phylogeny of *Pyrobombus* that includes molecular data and males from all taxa (including those treated as synonymies and known from one sex) occurs, the relationships of the taxa, and between the fauna of the Nearctic and Palearctic faunas will hold some unresolved issues. In the meantime, much work will be required to reassess collections to verify the identity of material presently identified as *B. lapponicus sylvicola*, and *B. melanopygus* from northern North America to facilitate conservation assessments (e.g., Hatfield et al. 2014; Canadian Endangered Species Conservation Council 2016) and studies of distribution (e.g., Williams et al. 2014; Sikes and Rykken 2020).

## Supplementary Material

XML Treatment for Bombus (Pyrobombus) johanseni

## Footnotes

The type locality of B. johanseni is in northern Nunavut; Northwest Territories was originally indicated by Sladen (1919) (Fig. 2c), and subsequently by Sarazin (1986), but Nunavut officially separated from the Northwest Territories on 1 April 1999 via the Nunavut Act. Though the specimen is labelled as a “Lectotype” designated by H.E. Milliron 1960 (Fig. 2c), we cannot find any published account of this designation, though it was not required as Sladen (1919: 30g) clearly indicate the “type” by original designation as a single queen collected on 3 July 1916 [1915 in Sladen 1919), the only specimen with that collection information. Though Sladen (1919) originally described and treated his taxon as a variety of B. sylvicolas. str., which was done so by subsequent authors (e.g., Frison 1927a, 1927b; Hurd 1979) we consider this name valid as according to Article, 45.6.4.1 of the ICZN Code (International Commission on Zoological Nomenclature 1999), an infrasubspecific name (i.e., under Article 45.6.4) is considered subspecific from its original publication use if, before 1985, it was either adopted as the valid name of a species or subspecies, which was done so by Lutz and Cockerell (1920) who treated it as a subspecies of B. sylvicola.
